# The plastid genome sequence of *Neocinnamomum delavayi* (Lec.) Liou

**DOI:** 10.1080/23802359.2019.1679051

**Published:** 2019-10-23

**Authors:** Shulin Ren, Yu Song, Meili Zhao, Wenbin Xu

**Affiliations:** a Center for Integrative Conservation, Xishuangbanna Tropical Botanical Garden, Chinese Academy of Sciences, Yunnan, China;; b Wuhan Botanical Garden, Chinese Academy of Sciences, Wuhan, China

**Keywords:** *Neocinnamomum*, chloroplast, phylogenetic analysis

## Abstract

*Neocinnamomum delavayi* (Lec.) Liou is a kind of medicinal plants belonging to the genus *Neocinnamomum* H. Liu, but is often confused with *N. mekongense* (Hand.-Mazz.) Kosterm. Here, the complete plastid sequence of the *N. delavayi* was determined. The length of the plastid genome is 150,584 bp with overall AT content of 61%. It exhibited a typical quadripartite structure comprising a large single copy region (LSC) of 91,887 bp, a small single copy region (SSC) of 18,443 bp, and a pair of inverted repeat regions (IRs) of 20,262 bp each. Maximum likelihood phylogenetic analysis with GTR + F+R2 model was performed using eighteen complete plastomes of the Lauraceae, which strongly supports the relationships: sisterhood of the *N. delavayi* and a clade containing *N. mekongense* and *N. lecomtei* Liou.

*Neocinnamomum delavayi* (Lec.) Liou, a widely distributed species in Sichuan and Yunnan of SW China, was assigned to the genus *Neocinnamomum* H. Liu in the family Lauraceae (http://foc.iplant.cn/). In China, *N. delavayi* is known as a kind of herbal medicine, whose leaves have long been used to cure the haemorrhage and rheumatism sufferers (Jiangsu new medical college 1977). It was also reported that its stem contains bioactive compounds such as sesquiterpenoid, flavonoid, and sterol (Yang et al. 2015). However, the morphological traits differ relatively little between *N. delavayi* and *N. mekongense* (Hand.-Mazz.) Kosterm and largely distinguished with silvery, sericeous hairs on the branchlets (Kostermans^1974^; Li et al. 1984). Wang et al. (2010) found that *N. delavayi* was located in a polytomy clade with three *N. mekongense* samples, suggesting that several molecular markers were limited to differentiate *N. delavayi* and *N. mekongense*. To distinguish *Neocinnamomum* species, it is necessary to perform high-throughput sequencing approaches.

Silica-gel dried leaves were sampled from a single individual growing in Wuhan Botanical Garden, Chinese Academy of Sciences (CAS) (Hubei, China; Long. 114.4234 E, Lat. 30.5444 N, 23 m). The specimens were deposited at the Biodiversity Research Group of Xishuangbanna Tropical Botanical Garden (XTBG) (Accession Number: XTBG-BGR-SY35377). Genomic DNA was extracted using a modified CTAB protocol (Doyle and Dickson 1987). The whole plastid genome was sequenced following Yang et al. (2014), and their nine universal primer pairs were used to perform long-range PCR for next-generation sequencing (Yang et al. 2014). The sequenced reads were assembled using GetOrganelle software (Jin et al. 2018). The contigs were aligned using the available plastid genome of *N. lecomtei* Liou (Song et al. 2017) and annotated in Geneious version 8.1.3 (Kearse et al. 2012).

**Figure 1.**
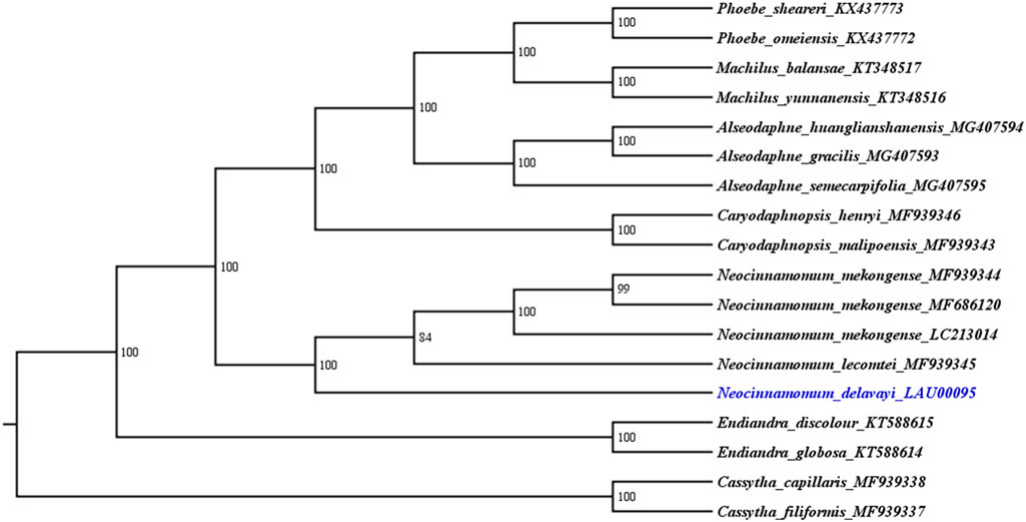
The ML phylogenetic tree for *N. delavayi* based on other 17 species (3 in *Alseodaphne*, 2 in *Caryodaphnopsis*, 2 in *Cassytha*, 2 in *Endiandra*, 2 in *Machilus*, 4 in *Neocinnamomum*, and 2 in *Phoebe*) plastid genomes.

The plastid genome of *N. delavayi* (LAU00095), with a length of 150,854 bp, was the largest among the two reported plastid genomes of *Neocinnamomum* species, was 16 bp and 12 bp larger than that of *N. lecomtei* (150,838 bp, MF939345) and *N. mekongense* (150,842 bp, MF939344), respectively. Its AT content is 61%. The plastid genome of *N. delavayi* includes double 20,262 bp inverted repeats (IRs) separated by a large single-copy region (LSC) and a small single-copy region (SSC) of 91,887 bp and 18,443 bp, respectively. There are 128 genes, including 84 protein-coding genes, 36 tRNAs, and eight rRNAs in the plastid genome of *N. delavayi*. Among these genes, 15 of which are duplicated in IRs, while 113 own single copy, which was commonly observed in other *Neocinnamomum* species (Song et al. 2019).

To confirm the relationships among *N. delavayi*, *N. lecomtei*, and *N. mekongense*, a maximum likelihood (ML) analysis was used to reconstruct the plastid genome phylogeny relationship among the eighteen species by the RAxML software (Stamatakis 2006) with 1000 bootstraps under the GTR + F+R2 substitution model. The phylogenetic tree with 84–100% bootstrap values at each node supports the relationships that a sisterhood of the *N. mekongense* and *N. lecomtei*, followed by N. delavayi (Figure 1).

## Data Availability

The plastome data of the *N. delavayi* will be submitted to Lauraceae Chloroplast Genome Database (https://lcgdb.wordpress.com). Accession numbers are LAU00095.
